# Association between intimate partner violence and nutritional status of married Nepalese women

**DOI:** 10.1186/s41256-022-00248-0

**Published:** 2022-05-18

**Authors:** Arun Chaudhary, Janet Nakarmi, Annekathryn Goodman

**Affiliations:** 1 Center for Sustainable Development Research, Kathmandu, Nepal; 2 Strength and Serenity: Global Initiative to End Gender-Based Violence, Boston, MA USA; 3 Department of Mathematics, University of Central Arkansas, Conway, AR USA; 4 Harvard Medical School, Boston, MA USA; 5 Division of Gynecologic Oncology, Massachusetts General Hospital, Boston, MA USA

**Keywords:** Intimate partner violence, Domestic violence, Gender-based violence, Violence against women, Sexual violence, Physical violence, Emotional violence, Nutrition, Women’s nutritional status

## Abstract

**Background:**

Intimate partner violence (IPV) is physical, sexual, or psychological harm perpetrated by a spouse or an intimate partner. Its detrimental effects on women’s physical, mental, sexual, and reproductive health are well-documented. However, its impact on nutritional status is not well-studied, and previous studies have led to contradictory findings. This study aimed to explore the association between intimate partner violence and the nutritional status of married Nepalese women.

**Methods:**

The study used the 2016 Nepal Demographic Health Survey data, which employed a modified version of the Conflict Tactics Scale to determine women’s exposure to IPV. Anemia and low body mass index (BMI) were used as proxies of nutritional status. Multinomial regression was used to analyze the relationship between BMI and IPV; multivariable logistic regression was used to analyze the association between anemia and IPV.

**Results:**

The prevalence of underweight, overweight/obesity, and anemia were respectively 13.9%, 25.1%, and 38.7%. The prevalence of physical, sexual, and emotional IPVs experienced in the preceding year were respectively 9.8%, 4.6%, and 7.6%. Likewise, the prevalence of lifetime physical, sexual, emotional, and controlling behavior IPVs were respectively 21.8%, 7.4%, 12.3%, and 32.1%. The low intensity of emotional IPV (AOR 1.62; CI: 1.02–2.56) and moderate intensity of physical IPV (AOR 3.70; CI: 1.64–8.35) experienced in the preceding year, and low intensity of lifetime emotional IPV (AOR 1.69; CI: 1.11–2.58) were associated with an increased risk of overweight/obesity. Moderate intensity of sexual IPV (AOR 2.59; CI: 1.099–6.108) experienced in the preceding year was associated with an increased risk of underweight BMI. The low intensity of lifetime controlling behavior (AOR1.25; CI: 1.03–1.53) was associated with an increased risk of anemia.

**Conclusions:**

Emotional and Physical IPVs are significantly associated with an increased risk of overweight/obesity. Sexual IPV is significantly associated with an increased risk of underweight BMI, and controlling behavior is significantly associated with an increased risk of anemia. Seeking help could offset the detrimental effects of IPV; therefore, IPV screening should be a part of regular healthcare assessment for married women, and appropriate rehabilitation should be offered to IPV survivors.

## Background

Intimate partner violence (IPV) is well recognized as a human rights violation and a serious global health issue. IPV is physical, sexual, or psychological harm caused by a spouse or an intimate partner [1]. The United Nations General Assembly declared the elimination of violence against women was urgently needed to promote “equality, security, liberty, integrity, and dignity of all human beings” [2]. Despite international efforts to eliminate violence against women, IPV prevalence among women has barely declined: 33.33% of worldwide women in 1985 compared to 30% of women had experienced some form of IPV in 2017 [3, 4].

IPV has detrimental effects on women’s physical, mental, sexual, and reproductive health [5–7]. Physical health problems include injuries, gastrointestinal disorders, hypertension, chronic pain, seizures, and fainting. IPV is also the underlying cause of 40–60% of female murder cases in North America, and this proportion is expected to be larger in low-income countries [5]. Mental health consequences of IPV include depression, post-traumatic stress disorders, suicide, insomnia, anxiety, social dysfunction, eating disorders, and substance abuse [5, 8].

IPV is also associated with an increased risk of reproductive health problems such as unintended pregnancy, sexually transmitted diseases, gynecological disorders, and adverse birth outcomes [5–7]. IPV-associated gynecological problems include vaginal bleeding, vaginal infection, reduced sexual desire, genital irritation, painful intercourse, urinary tract infections, and pelvic pain [5, 6].

Nepal’s gender inequality index (0.476) ranks 115 among 189 countries, which indicates severe gender inequality in the country [9]. Patriarchy is entrenched in Nepal’s sociocultural norm: practices like dowry, marginalization of women, female child marriages, acceptance of violence against women are common practices [10]. The Government of Nepal formulated Domestic Violence Offence and Punishment Act in 2009, recognizing violence against women as a punishable crime [11]. Despite the stringent law, about one-fourth of Nepalese women continue to experience IPV each year, and 66% of IPV survivors do not seek help to cope with violence [12].

About 15% of non-pregnant Nepalese women of reproductive age are underweight; 19% are overweight; 5% are obese; 20% are anemic [13]. Nepalese women’s age, employment, residence, wealth, breastfeeding practice, and empowerment status are associated with their nutritional status [14–17]. Additional known risk factors of women’s poor nutritional status include pregnancy, smoking, gender disparity, low literacy, unemployment, low empowerment, and food insecurity [18–22].

Withholding of food is a form of physical violence, which would have direct adverse effect on women’s nutritional status [23]. Inadequate food portions for men in food insecure families can trigger violence, so women may avoid eating enough to prevent violent encounters [23]. Inadequate calorie intake and micronutrients deficiency are known risk factors of low BMI and anemia. IPV can also trigger health conditions and behaviors that indirectly affect women’s nutritional status. IPV survivors are prone to psychological stress, anxiety, depression, smoking, and drinking problems [5, 8], which can degrade one’s nutritional status. Psychological stress heightens cellular oxidative stress, making body tissues prone to prematurely degeneration, potentially leading to low body mass index or hemolytic anemia [24–26]. Depression is closely associated with loss of appetite, a clinical symptom highly correlated with low BMI [27, 28]. There is also evidence that anxiety disorder can lead to overeating, a psychological phenomenon that can increase BMI [29]. These are the potential pathways through which IPV may cause underweight, overweight/obese BMI, or anemia.

A study in Bangladesh found that survivors of physical IPV (PIPV) and sexual IPV (SIPV) have an increased risk of being underweight [30]. A similar study in India found that physical domestic violence increases women’s risk of anemia and underweight BMI [31]. Another study in Nepal found that men’s controlling behavior (CBIPV) is associated with an increased risk of anemia, and PIPV is associated with decreased risk of overweight/obesity [32]. Previous studies have either analyzed the IPV types separately or failed to examine all four IPV types, so a particular IPV type could have masked the effect of excluded IPV types. The previous studies have also failed to adjust their analyses for whether IPV survivors sought help to cope with IPV incidences, which would offset its health effects. Former studies have not differentiated the severity of various IPV forms or accounted for their frequency, which would determine the exposure dosage more accurately. Food security and household wealth are well-known determinants of nutritional status, and menstruation can cause anemia due to blood loss. Rahman et al., Ackerson et al., and Adhikari et al. did not adjust their analyses for food security or menstruation. Rahman et al. did not adjust their analyses for household wealth also, which could have skewed their results. This study addressed the deficiencies in previous studies and aimed to explore the association between intimate partner violence and the nutritional status of married Nepalese women.

## Methods

This study is a cross-sectional study using nationally representative 2016 Nepal Demographic Health Survey (NDHS) data. Married Nepalese women of age 15 to 49 years are the subjects of this study. Divorcees do not experience IPV after separation, making their exposure to IPV quite different from partnered women, so they were excluded from this study. As pregnancy can confound the estimation of actual body mass index (BMI) and hemoglobin concentration, women who were pregnant or had given birth in the preceding two months were also excluded from this study. The 2016 NDHS used a multistage stratified cluster sampling method and employed the probability proportional to size method to select sampling units [12]. The total number of women whose weight, height, and hemoglobin were measured, who completed the domestic violence module and met this study’s inclusion and exclusion criteria was 3422. Forty-three of them had data missing for the study’s primary exposure or outcomes, so the final study sample comprised 3,379 women.

The 2016 NDHS used a modified version of the Conflict-Tactics Scale to collect information regarding IPV experience [12]. Four types of IPV—physical, sexual, emotional, and controlling behavior—were analyzed in this study. Except for the controlling behavior, each IPV type was analyzed as lifetime exposure (IPVL) and the preceding year experience (IPVY). Data for the preceding year’s controlling behavior was unavailable, so it was only analyzed as a lifetime experience. This study examined seven forms of physical IPV (PIPV), three forms of sexual IPV (SIPV), three forms of emotional IPV (EIPV), and five forms of controlling behavior IPV (CBIPV). Each IPV form corresponded to the original IPV survey question, which are listed in “Appendix 1”.

The severity of different PIPV forms was determined based on the scale developed by Marshal [33], which is also followed by the US Centers for Disease Control and Prevention [34]. The PIPV forms were categorized as mildly, moderately, or severely harmful. Although Marshall did not explicitly determine the severity of SIPV forms, this study’s participants perceived all forms of SIPV as physically and emotionally very harmful, so all SIPV forms were categorized as severely harmful in this study’s analysis. The severity of EIPV and CBIPV forms were also not specified in the scale developed by Marshall, but their nature closely resembles that of symbolic violence included in the study, which the study participants perceived as less harmful. Therefore, all EIPV and CBIPV forms except for ‘threaten to hurt or harm’ were categorized as mildly severe in this study’s analysis. ‘Threaten to hurt or harm’ was classified as moderately harmful because it is the only EIPV form that includes an explicit threat. As detailed in “Appendix 1”, scores of 1, 2, and 3 were respectively assigned to mildly, moderately, and severely harmful IPV forms.

The 2016 NDHS did not collect data on the frequency of lifetime IPV experience, which would have been prone to recall bias anyways if it was collected. If an IPVL form was ever experienced, a frequency score of 1 was assigned, otherwise a score of 0 was assigned. Likewise, scores of 0, 1, and 2 were respectively assigned if an IPVY form was not experienced in the preceding year, experienced sometimes, or often in the past year. The severity and frequency scores were multiplied to compute an IPV score, as detailed in “Appendix 1”. The individual scores were then added to calculate a total score for each IPV type. Finally, those with a total IPV score of 0 was categorized as none, and the remaining range of IPV scores was divided into three inter-quartiles, where the first interquartile was categorized as mildly intense, second interquartile as moderately intense, and the third interquartile as highly intense (as detailed in “Appendix 2”).

Blood hemoglobin concentration and body mass index (BMI) were used as proxies of nutritional status. As high altitude and smoking can elevate hemoglobin concentration, adjusted hemoglobin concentration was used to determine the anemic status [35]. The World Health Organization’s guidelines for international classification of adult BMI were used to determine underweight or overweight/obese BMI [36]. A BMI of less than 18.5 kg/m^2^ was categorized as underweight; 25.0 kg/m^2^ or more was categorized as overweight/obese; 18.5 to 24.9 kg/m^2^ was categorized as normal. The WHO’s recommended hemoglobin cutoff point for non-pregnant women was used to determine anemia [37]. Women with a hemoglobin concentration less than 120 g/liter were classified as anemic.

Other covariates analyzed in this study included whether women ever sought help to cope with IPV, if injuries were ever suffered from IPV incidences, age, place of residence, education level, ethnicity, employment status, family size, household wealth, sex of the household head, number of years lived with the partner, partner’s age, women’s decision-making score, household’s food insecurity, number of children under the age of five, and time length since last menstruation. Although smoking is a known determinant of weight, it was not treated as a confounder because IPV has been proven to cause smoking [38], making it an intermediate variable that does not have to be treated as a confounder. Although most variables were readily available in the 2016 NDHS dataset, household food insecurity and women’s decision-making role indices were calculated using relevant variables. An index for women’s decision-making role was calculated based on Data for Impact Project’s guidelines using three indicators: decisions about healthcare, large household purchases, and family or relative visits [39]. The Household Food Insecurity Access Score was calculated based on the Food and Nutrition Technical Assistance Project guidelines [40]. While the decision-making score ranges from 0 to 3, the food insecurity score ranges from 0 to 27.

The statistical software R was used for the statistical analysis of this study. A *p*-value of 0.05 (95% CI) was used to determine the statistical significance of variables in this study’s analyses. Prevalence of outcomes of interests—underweight, overweight/obese, and anemia—were determined across various sociodemographic strata.

The outcome variable BMI was measured as a multinomial outcome (normal, underweight, or overweight/obese), and the hemoglobin level was analyzed as a dichotomous outcome (non-anemic or anemic). Two multinomial logistic regression models were fitted for BMI, one for IPVL and the other for IPVY. Likewise, two multivariable logistic regression models were fitted for anemia, one for IPVL and the other for IPVY. A method called ‘purposeful selection of covariates’ was used to fit all regression models [41].

## Results

### Characteristics of the subjects

Among 3379 women included in the study, 13.5% had experienced at least one type of IPV in the preceding year. The corresponding estimate for lifetime IPV experience was 42% (inclusive of the controlling behavior IPV). The prevalence of physical (PIPVY), sexual (SIPVY), and emotional IPVs (EIPVY) in the preceding year were respectively 9.8%, 4.6%, and 7.6%. Likewise, the prevalence of lifetime physical (PIPVL), sexual (SIPVL), emotional (EIPVL), and controlling behavior IPVs (CBIPVL) were 21.8%, 7.4%, 12.3%, and 32.1% respectively.

13.9% of women were underweight, 25.1% were overweight/obese, and 38.7% were anemic. The prevalence of underweight and overweight/obese BMIs and anemia across various sociodemographic characteristics are listed in Table 1.

**Table 1:** Prevalence of BMI statuses and anemia across sociodemographic characteristics

Characteristics	Categories	Total (n)	Prevalence of nutritional statuses (row %)		
			Overweight/obese	Underweight	Anemia
Age	15–19	161	5.0	24.8	38.5
	20–24	524	12.8	17.6	40.5
	25–29	676	23.7	14.6	40.2
	30–34	692	29.3	10.8	39.7
	35–39	591	30.8	10.7	36.9
	40–44	421	30.4	14.7	37.1
	45–49	314	31.5	12.1	36.0
Religion	Hindu	2985	24.2	14.0	39.4
	Buddhist	149	38.9	6.0	23.5
	Muslim	121	16.5	28.1	51.2
	Kirat	43	44.2	4.7	18.6
	Christian	81	35.8	8.6	34.6
Ethnicity	Terai Dalit	125	13.6	34.4	60.0
	Hill Brahmin	396	30.6	8.8	37.1
	Hill Chhetri	765	18.7	13.6	34.1
	Terai Brahmin/Chhetri	53	39.6	13.2	62.3
	Madhesi	379	14.5	23.5	53.6
	Hill Dalit	355	26.2	11.8	30.1
	Newar	121	47.9	4.9	20.7
	Hill Janajati	716	36.2	6.4	25.1
	Terai Janajati	334	15.9	18.3	62.3
	Muslim	121	16.5	28.1	51.2
	Other	14	50.0	14.3	50.0
Residence	Urban	2119	29.3	12.5	38.0
	Rural	1260	18.0	16.2	39.8
Education	No education	1443	18.4	18.0	40.5
	Primary	609	27.6	12.0	37.9
	Secondary	967	29.3	10.7	37.0
	Higher	360	36.4	9.2	37.2
Household Wealth Index	Poorest	772	11.7	20.2	32.8
	Poorer	764	16.8	14.4	36.4
	Middle	752	24.1	12.8	43.0
	Richer	566	32.2	12.7	45.9
	Richest	525	50.7	6.7	37.0
Employment	Yes	2161	24.4	13.1	36.4
	No	1218	26.3	15.2	42.9
Decision-making score	0	764	14.3	19.6	40.8
	1	547	21.4	15.5	36.0
	2	684	28.2	12.4	37.3
	3	1384	30.9	10.8	39.3
Total (N)		3379	25.1	13.9	38.7

**Fig. 1:**
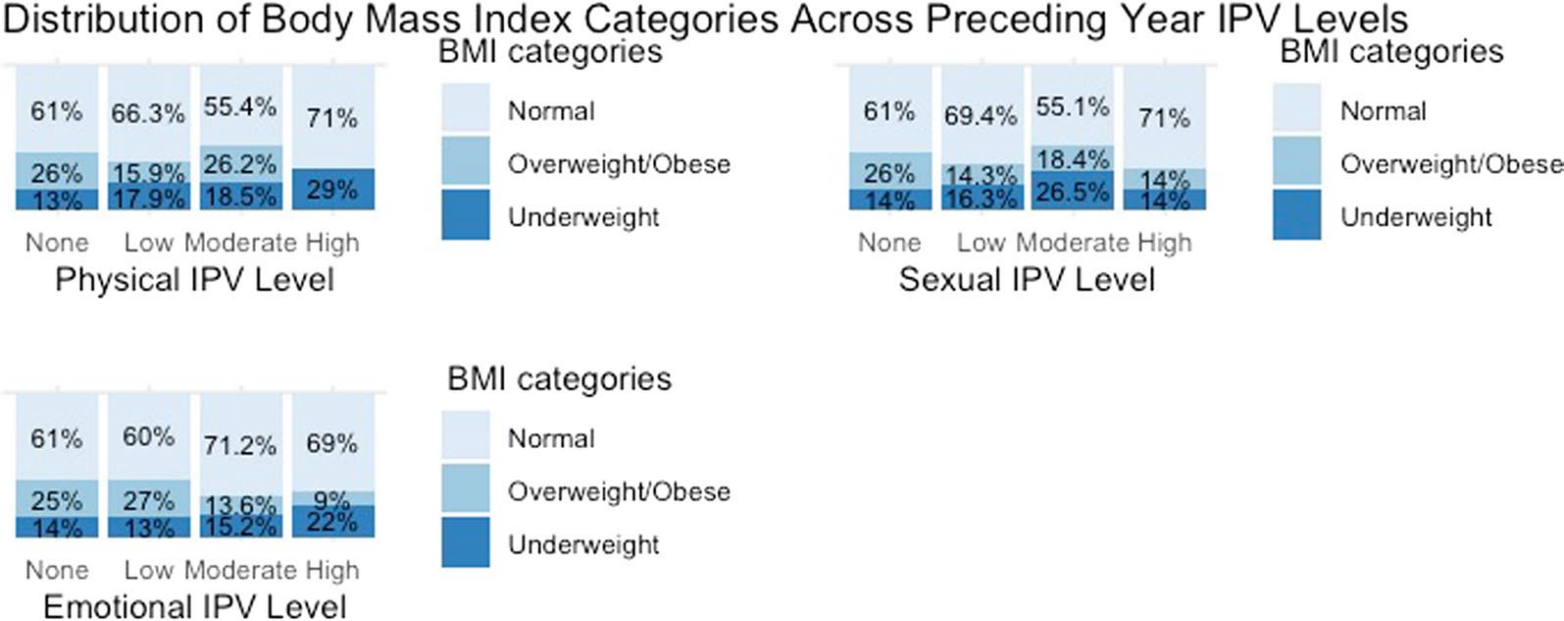
Distribution of BMI categories across preceding year IPV Intensity Levels

**Fig. 2:**
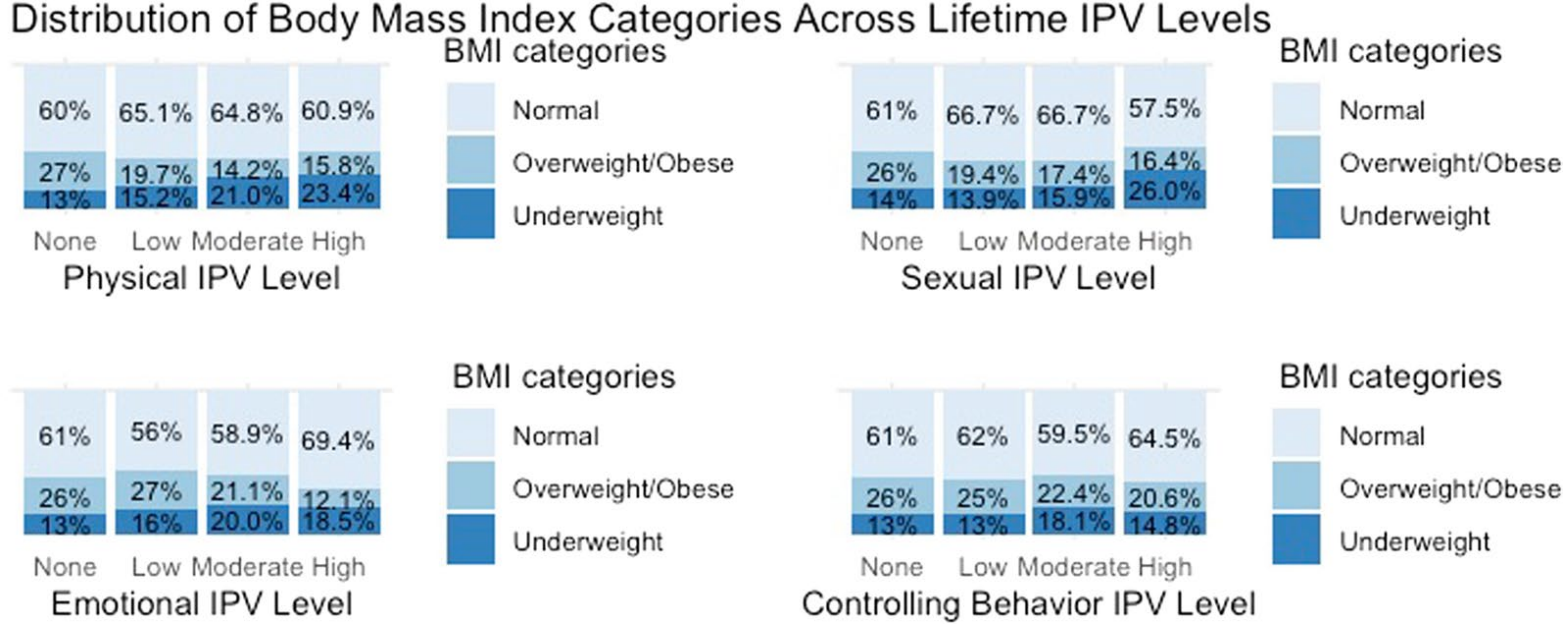
Distribution of BMI categories across lifetime IPV intensity levels

The distribution of overweight/obese and underweight BMI statuses across IPVY and IPVL intensity levels are graphically represented in Figs. 1 and 2, respectively.

### Preceding year IPV experience & body mass index (regression model 1)

In the adjusted model for IPVY, only low intensity of EIPVY and moderate intensity of PIPVY were statistically significant for overweight/obese BMI status. Compared to women who had not experienced any EIPVY, those who had experienced low intensity of EIPVY had 1.62 (CI: 1.02–2.56) times higher odds of being overweight/obese. Similarly, those who had experienced the moderate intensity of PIPVY had 3.70 (CI: 1.64–8.35) times higher odds of being overweight/obese. On the other hand, women who had experienced the moderate intensity of SIPVY had 2.59 (CI: 1.099–6.108) times higher odds of being underweight than those who had not experienced SIPVY. The results of multinomial regression models for IPVY and BMI are listed in Table 2.

**Table 2:** Multinomial logistic regression models: intimate partner violence and body mass index

		Preceding Year Intimate Partner Violence (Model 1)					Lifetime Intimate Partner Violence (Model 2)			
		Overweight			Underweight		Overweight		Underweight	
Covariates	Categories	AOR		AOR 95% CI	AOR	AOR 95% CI	AOR	AOR 95% CI	AOR	AOR 95% CI
Emotional IPV	None (Reference)									
	Low	1.617*		(1.018–2.566)	0.655	(0.373–1.150)	1.694*	(1.114–2.576)	1.153	(0.714–1.861)
	Moderate	0.655		(0.275–1.561)	0.660	(0.295–1.474)	1.159	(0.618–2.173)	1.720	(0.912–3.244)
	High	0.644		(0.160–2.591)	1.002	(0.297–3.381)	0.700	(0.348–1.406)	1.203	(0.645–2.244)
Physical IPV	None (Reference)									
	Low	0.972		(0.600–1.575)	1.101	(0.699–1.733)	1.874	(0.726–4.836)	0.737	(0.257–2.115)
	Moderate	3.700**		(1.640–8.348)	1.131	(0.480–2.667)	1.524	(0.552–4.212)	1.330	(0.451–3.919)
	High	NR		NR	1.278	(0.336–4.862)	2.443	(0.853–7.002)	1.636	(0.545–4.913)
Sexual IPV	None (Reference)									
	Low	0.713		(0.353–1.440)	1.077	(0.571–2.030)	1.341	(0.665–2.706)	0.723	(0.347–1.508)
	Moderate	0.895		(0.348–2.304)	2.591*	(1.099–6.108)	0.844	(0.384–1.854)	1.121	(0.524–2.400)
	High	1.014		(0.075–13.725)	0.411	(0.039–4.352)	0.919	(0.392–2.158)	1.601	(0.747–3.433)
Control behavior	None (Reference)									
	Low	NA		NA	NA	NA	0.987	(0.772–1.261)	0.822	(0.612–1.104)
	Moderate	NA		NA	NA	NA	1.335	(0.966–1.846)	1.015	(0.718–1.435)
	High	NA		NA	NA	NA	1.109	(0.646–1.905)	0.632	(0.347–1.153)
Ever sought help	Never experienced IPV (Reference)									
	No	0.559		(0.298–1.049)	1.191	(0.649–2.184)	0.570	(0.303–1.072)	1.224	(0.667–2.247)
	Yes	0.619		(0.322–1.188)	0.757	(0.383–1.496)	0.601	(0.312–1.157)	0.708	(0.356–1.408)
Experienced injury	Never experienced IPV (Reference)									
	No	1.337		(0.683–2.619)	0.936	(0.488–1.795)	0.681	(0.220–2.111)	0.969	(0.295–3.187)
	Yes	0.674		(0.318–1.431)	0.937	(0.461–1.905)	0.358	(0.100–1.282)	0.665	(0.181–2.443)
Age (Yrs.)		1.047***		(1.035–1.059)	0.991	(0.978–1.004)	1.048***	(1.037–1.060)	0.989	(0.976–1.003)
Place	Rural (Reference)									
	Urban	1.786***		(1.463–2.182)	0.816	(0.660–1.030)	1.783***	(1.467–2.178)	0.821	(0.659–1.031)
Ethnicity	Terai Dalit (Reference)									
	Hill Brahmin	1.119		(0.601–2.072)	0.233***	(0.133–0.396)	1.155	(0.619–2.149)	0.228***	(0.131–0.395)
	Hill Chhetri	0.971		(0.531–1.775)	0.241***	(0.149–0.382)	0.991	(0.539–1.808)	0.235***	(0.146–0.376)
	Terai Brahmin/Chhetri	1.593		(0.684–3.709)	0.501	(0.186–1.290)	1.554	(0.667–3.617)	0.499	(0.192–1.303)
	Madhesi	0.629		(0.331–1.196)	0.658	(0.409–1.046)	0.656	(0.344–1.244)	0.653	(0.406–1.044)
	Hill Dalit	2.161*		(1.152–4.052)	0.226***	(0.131–0.380)	2.231*	(1.189–4.198)	0.218***	(0.127–0.375)
	Newar	2.252*		(1.119–4.531)	0.183***	(0.071–0.465)	2.293*	(1.140–4.616)	0.172***	(0.067–0.441)
	Hill Janajati	2.577**		(1.423–4.669)	0.141**	(0.085–0.237)	2.634**	(1.446–4.752)	0.139***	(0.082–0.233)
	Terai Janajati	0.779		(0.408–1.488)	0.443**	(0.263–0.707)	0.785	(0.409–1.494)	0.442**	(0.268–0.726)
	Muslim	0.845		(0.393–1.818)	0.924	(0.516–1.637)	0.846	(0.392–1.817)	0.938	(0.521–1.668)
	Other	3.044		(0.805–11.508)	0.755	(0.137–4.167)	3.269	(0.854–12.386)	0.812	(0.146–4.470)
Wealth Index	Poorest (Reference)									
	Second Poorest	1.511*		(1.103–2.071)	0.576***	(0.432–0.782)	1.511*	(1.104–2.077)	0.586***	(0.436–0.791)
	Middle	2.401***		(1.757–3.282)	0.441***	(0.322–0.621)	2.388***	(1.746–3.269)	0.444***	(0.318–0.617)
	Second Richest	3.867***		(2.783–5.374)	0.453***	(0.312–0.659)	3.835***	(2.763–5.349)	0.453***	(0.311–0.660)
	Richest	7.780***		(5.520–10.964)	0.283***	(0.181–0.463)	7.846***	(5.553–11.061)	0.292***	(0.182–0.467)
Food Insecurity Score			0.968*	(0.943–0.993)	0.994	(0.969–1.020)	0.967*	(0.942–0.992)	0.995	(0.970–1.021)
Decision Makin Score			1.147***	(1.058–1.243)	0.887**	(0.815–0.970)	1.148***	(1.059–1.244)	0.893**	(0.818–0.974)

*NR* not reported because of insufficient observations, *NA* not applicable

Significance intensity *0.05, **0.01, ***0.001

Age, residence, ethnicity, wealth index, food insecurity, and decision-making scores were statistically significant for overweight/obese BMI. Being older (AOR 1.05; CI: 1.04–1.06) and residence in urban areas (AOR 1.79; CI: 1.46–2.18) increased the odds of being overweight/obese. Ethnic groups hill Dalit (AOR 2.16; CI: 1.15–4.05), Newar (AOR 2.25; CI: 1.12–4.53), and hill Janajati (AOR 2.58; CI: 1.42–4.67) had higher odds than terai Dalits of being overweight/obese. Compared to women from the poorest households, women from the second poorest households (AOR 1.51; CI: 1.10–2.07), the middle class (AOR 2.40; CI: 1.76–3.28), the second richest (AOR 3.87; CI: 2.78–5.37), and the richest households (AOR 7.78; CI: 5.52–10.96) had increased odds of being overweight/obese. While a higher food insecurity score (AOR 0.97; CI: 0.94–0.99) decreased the odds of being overweight/obese, women’s higher decision-making score (AOR 1.15; CI: 1.06–1.24) increased it.

In case of underweight BMI status, ethnicity, household’s wealth, and women’s decision-making score were statistically significant. Hill Brahmins (AOR 0.23; CI: 0.13–0.40), hill Chhetris (AOR 0.24; CI: 0.15–0.38), hill Dalits (AOR 0.23; CI: 0.13–0.38), Newars (AOR 0.18; CI: 0.07–0.47), hill Janajatis (AOR 0.14; CI: 0.09–0.24), and terai Janajatis (AOR: 0.44; CI: 0.26–0.71) had lower odds of being underweight than terai Dalits. The second poorest (AOR 0.58; CI: 0.43–0.78), the middle class (AOR 0.44; CI: 0.32–0.62), the second richest (AOR 0.45; CI: 0.31–0.66), and the richest (AOR 0.29; CI: 0.18–0.46) women had lower odds of being underweight compared to the poorest women. Higher decision-making score decreased the odds of being underweight (AOR 0.89; CI: 0.82–0.97).

### Lifetime IPV experience & body mass index (regression model 2)

In the adjusted model for lifetime IPV experience, only low intensity of EIPVL was statistically significant for overweight/obese BMI. Women who had experienced low intensity of EIPVL had 1.69 (CI: 1.11–2.58) times higher odds of being overweight/obese compared to those who had never experienced EIPVL. None of the IPVL types was significantly associated with underweight BMI status. Age, place of residence, ethnicity, wealth index, food insecurity, and decision-making scores were significantly associated with BMI. The magnitude and direction of their relationship with overweight/obese and underweight BMI statuses nearly equaled the estimates for the IPVY discussed in the previous section. The results of the multinomial regression model of IPVL and BMI are listed in Table 2.

**Fig. 3:**
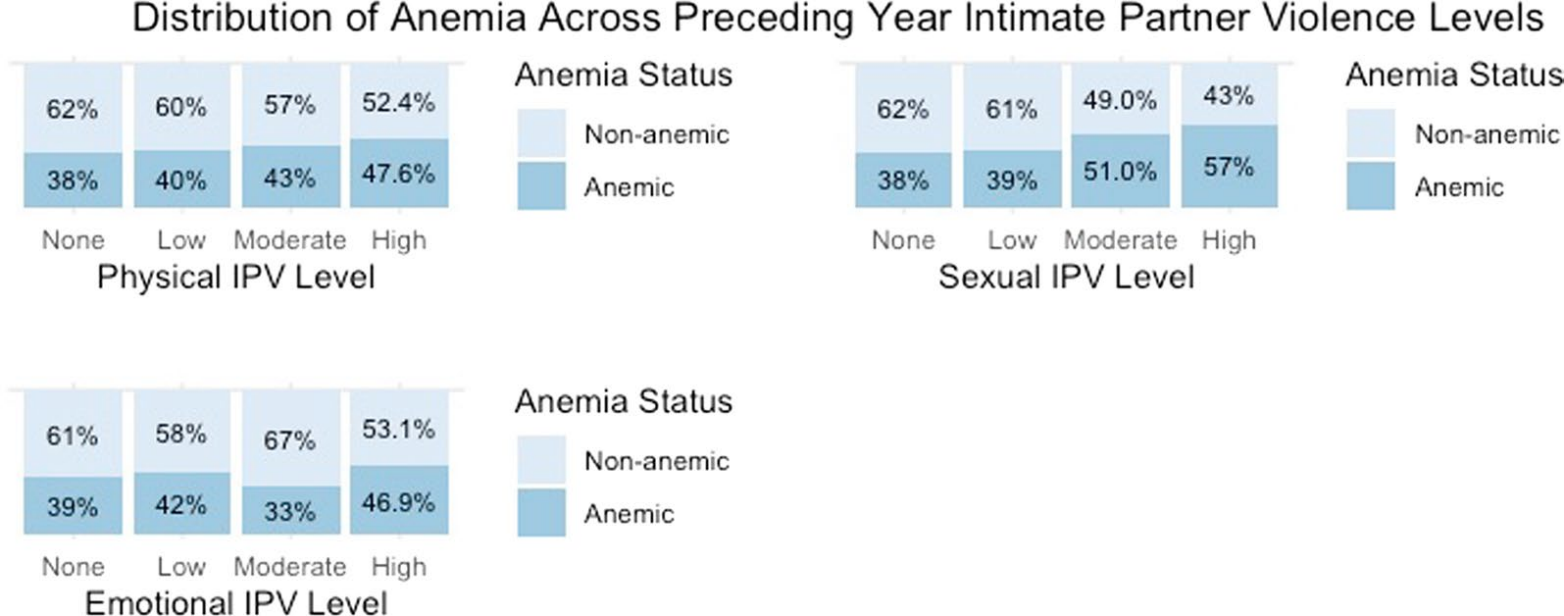
Distribution of anemic status across preceding year IPV intensity levels

**Fig. 4:**
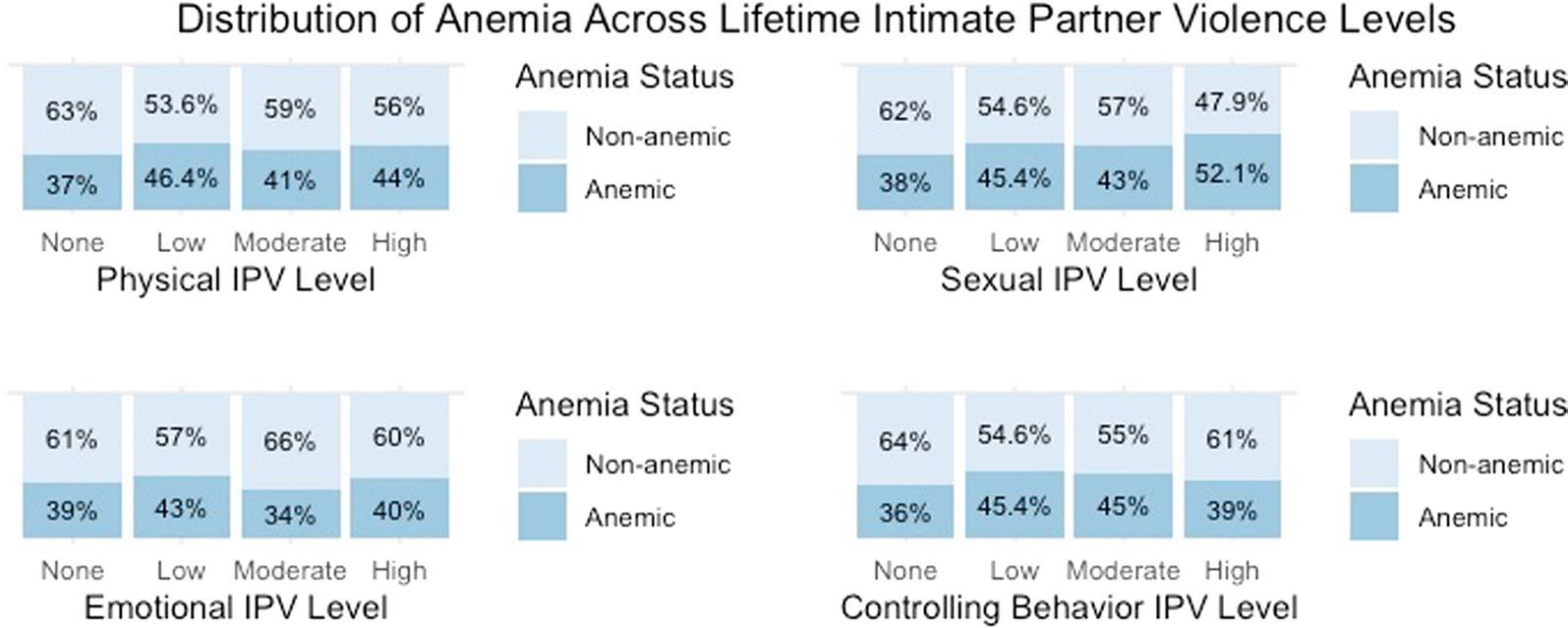
Distribution of anemic status across lifetime IPV intensity levels

The distribution of anemia across IPVY and IPVL intensity levels is graphically represented in Figs. 3 and 4, respectively.

### Preceding year IPV experience & anemia (regression model 3)

In the adjusted model for the IPVY, none of the IPVs was significantly associated with anemic status. However, IPV survivors who had not sought help had 1.64 (CI: 1.05–2.56) times higher odds of being anemic compared to those who had not experienced IPVY. The results of multivariable logistic regression models for IPVY and anemic status are detailed in Table 3.

**Table 3:** Multivariate logistic regression models: intimate partner violence and anemia

		Preceding year intimate partner violence (Model 3)		Lifetime intimate partner violence (Model 4)	
Covariates	Categories	Anemia AOR	AOR 95% CI	Anemia AOR	AOR 95% CI
Emotional IPV	None (Reference)				
	Low	0.941	(0.644–1.368)	0.967	(0.686–1.357)
	Moderate	0.629	(0.335–1.153)	0.670	(0.399–1.106)
	High	1.082	(0.414–2.744)	0.802	(0.493–1.295)
Physical IPV	None (Reference)				
	Low	0.805	(0.567–1.141)	0.928	(0.450–1.914)
	Moderate	0.989	(0.518–1.873)	0.926	(0.432–1.988)
	High	0.813	(0.247–2.603)	1.046	(0.477–2.297)
Sexual IPV	None (Reference)				
	Low	0.882	(0.543–1.422)	1.285	(0.760–2.163)
	Moderate	1.912	(0.960–3.816)	1.221	(0.685–2.168)
	High	1.567	(0.300–9.065)	1.800	(0.974–3.349)
Control behavior IPV	None (Reference)				
	Low	NA	NA	1.252*	(1.027–1.525)
	Moderate	NA	NA	1.123	(0.871–1.447)
	High	NA	NA	0.984	(0.641–1.499)
Ever sought help	Never experienced IPV (Reference)				
	No	1.641*	(1.048–2.564)	1.629*	(1.038–2.552)
	Yes	1.099	(0.679–1.764)	1.086	(0.670–1.746)
Experienced injury	Never experienced IPV (Reference)				
	No	0.816	(0.505–1.321)	0.762	(0.328–1.766)
	Yes	0.830	(0.491–1.402)	0.728	(0.287–1.842)
Ethnicity	Terai Dalit (Reference)				
	Hill Brahmin	0.447***	(0.291–0.681)	0.449***	(0.292–0.686)
	Hill Chhetri	0.398***	(0.265–0.592)	0.398***	(0.266–0.594)
	Terai Brahmin/Chhetri	1.175	(0.603–2.326)	1.138	(0.583–2.256)
	Madhesi	0.785	(0.515–1.191)	0.774	(0.507–1.173)
	Hill Dalit	0.329***	(0.212–0.505)	0.328***	(0.211–0.506)
	Newar	0.197***	(0.109–0.346)	0.200***	(0.110–0.352)
	Hill Janajati	0.258***	(0.171–0.386)	0.258***	(0.171–0.387)
	Terai Janajati	1.220	(0.792–1.871)	1.200	(0.779–1.840)
	Muslim	0.690	(0.412–1.151)	0.686	(0.409–1.146)
	Other	0.666	(0.212–2.093)	0.628	(0.199–1.980)
Wealth Index	Poorest (Reference)				
	Second Poorest	1.051	(0.837–1.319)	1.054	(0.839–1.323)
	Middle	1.256	(0.994–1.588)	1.235	(0.976–1.563)
	Second Richest	1.434**	(1.112–1.850)	1.397**	(1.082–1.804)
	Richest	1.017	(0.774–1.337)	1.005	(0.764–1.323)
Food Insecurity Score		1.003	(0.984–1.022)	1.001	(0.982–1.020)
Time Since Last Menstruation	One week (Reference)				
	Two weeks	0.905	(0.721–1.135)	0.909	(0.724–1.142)
	Three weeks	0.989	(0.784–1.250)	0.984	(0.778–1.244)
	Four weeks or more	0.856	(0.704–1.041)	0.851	(0.699–1.035)
No. of children under 5 Yrs		1.133**	(1.036–1.240)	1.131**	(1.033–1.238)

Significance intensity *0.05, **0.01 ***0.001

*NA* not applicable

Ethnicity, wealth index, and the number of children under five were statistically significant for anemic status. Hill Brahmins (AOR 0.45; CI: 0.29–0.68), hill Chhetris (AOR 0.40; CI: 0.27–0.59), hill Dalits (AOR 0.33; CI: 0.21–0.51), Newar (AOR 0.19; CI: 0.11–0.35), and hill Janajatis (AOR 0.26; CI: 0.17–0.39) had smaller odds of being anemic compared to terai Dalits. Women from the second richest households (AOR 1.43; CI: 1.11–1.85) were more likely to be anemic than those from the poorest households. The larger the number of women’s children under five years of age (AOR 1.13; CI: 1.04–1.24), the higher the odds of being anemic.

### Lifetime IPV experience & anemia (regression model 4)

In the case of IPVL, the low intensity of CBIPVL and whether IPV survivors sought help were significantly associated with anemic status. Compared to women who had had never experienced CBIPVL, those who had experienced the low intensity of CBIPVL were 1.25 (CI: 1.03–1.53) times more likely to be anemic. IPVL survivors who had never sought help had 1.63 (CI: 1.04–2.55) times higher chance of being anemic than those who had never experienced IPVL. Ethnicity, wealth index, and the number of children under five were statistically significant for anemic status. The magnitude and direction of their relationship with anemia nearly equaled the estimates for IPVY discussed in the previous section.

## Discussion

The AOR between high PIPVY intensity and overweight/obese BMI was not reported because the category lacked observations. EIPVY’s low intensity but not moderate or high intensity was significantly associated with overweight/obese BMI probably because a larger proportion of higher intensity EIPVY survivors had sought help against violence (low: 20.0%, moderate: 28.8%, high 37.5%). In the case of underweight BMI, only moderate SIVPY intensity was significantly associated probably because of the small number of observations in the high SIPVY intensity category (n = 7). EIPV most likely has a lasting effect on BMI, as suggested by the statistically significant relationship between the low intensity of EIPVY or EIPVL with overweight/obese BMI. In contrast, PIPV and SIPV most likely do not have a prolonged effect on BMI, the reason why PIPVY and SIPVY were statistically significant, but PIPVL and SIPVL were not statistically significant.

The IPVY was not significantly associated with anemia; however, low CBIPVL intensity was associated with an increased risk of anemia. A smaller proportion of low CBIPVL intensity survivors (6.1%) compared to moderate (12.5%) and high CBIPVL (27.1%) intensity survivors had sought help against IPV. It probably explains why low but not moderate or high CBIPVL intensity was significantly associated with anemia. Unlike IPV survivors who had sought help, those who had not sought help were associated with an increased risk of anemia. It could mean that the isolated effects of IPVY types were too small and could not be detected; however, their collective effect significantly affected anemia. It can also be inferred that seeking help offsets IPV’s increased risk of anemia. Help-seeking history’s effect modification of IPV could not be tested because there were not enough observations across various combinations of IPV intensities, anemic status, and ‘help-seeking history categories. Although the time since last menstruation was not significantly associated with anemia, our study retained it in the regression models because of the known relationship between menstrual bleeding and anemia [42].

A similar study in Bangladesh found that survivors of PIPV are 1.22 times (95% CI 1.02–1.46), survivors of SIPV are 1.1 times (95% CI 0.74–1.63), and survivors of both PIPV and SIPV are 1.24 times (95% CI 1.04–1.58) more likely to be underweight than women who have not experienced IPV [30]. Another study in India found that women who have experienced physical domestic violence are 1.27 times (95% CI 1.02–1.57) more likely to be severely anemic and 1.2 times (95% CI 1.06–1.35) more likely to be severely underweight [31]. A recent study in Nepal did not find a significant association between being underweight and any type of IPV [32]. However, Adhikari et al. found that controlling behaviors increased women’s odds of being anemic by 31% (95% CI = 1.11–1.54). The disagreements between our study’s findings and previous studies are most likely due to the differences in study methods.

One of the main strengths of this study is that it compares the effect of the preceding year and lifetime IPV experience on women’s nutritional status. Another strength of this study is that it has used an improved measurement of women’s exposure to IPV, compared to previous analyses that treats all IPV forms as equally severe and do not account for frequency of IPV forms. Although this study differentiates the IPV intensity based on unvalidated method, it potentially produces a more accurate analysis compared to previous studies and can contribute to the future discussion on improvement of IPV measurement. Unlike the previous studies, the inclusion of all IPV types in a single regression model adjusted their effects and isolated the true magnitude of the association between a specific IPV type and nutritional status.

IPV can take many forms, and the ones measured by the 2016 NDHS and included in this study are not comprehensive. IPV is a sensitive topic and could have been underreported; therefore, this study may have failed to detect some positive cases of IPV. As this study excluded divorcees and pregnant women, findings of this study are only applicable to married, non-pregnant women. The original survey question asked if women ever sought help for any IPV, making it more appropriate for the lifetime analysis. This study assumes that women who had felt empowered enough to seek help more than a year ago would also seek help in the preceding year; therefore, it was included in IPVY analyses. The IPV scoring and intensity catergorization method used in this study is not a validated method, so developing an improved IPV measurement based on this study’s methodology is recommended for future studies. A prospective study investigating the span of IPV’s harmful effect is also recommended, which would more precisely compare the effects of IPVY and IPVL. As statistically significant IPVs observed in lifetime IPV analysis were also observed in the preceding year IPV analysis but not vice versa, the preceding year IPV is likely the more accurate method of analyzing IPV.

## Conclusions

Only some IPV types have a statistically significant effect on nutritional status. While emotional and physical IPVs increase the risk of overweight/obese BMI, sexual IPV increases the risk of underweight BMI. Unlike PIPV and SIPV, EIPV’s effect on BMI lasts for more than a year and therefore has a more enduring effect. Controlling behavior is the only IPV type that has a significant effect on anemic status. Although other IPV types do not significantly affect the anemic status on their own, their collective effect could be significantly associated with anemia. Wealth index, decision-making role, food security, and ethnicity, all indicators of women’s socioeconomic status, are significant determinants of their nutritional status. Seeking help to cope with IPV incidences could offset its detrimental effect on nutritional status. Therefore, IPV screening should be a part of regular healthcare assessment for married women, and appropriate rehabilitation should be offered to IPV survivors.

The abbreviations used in the manuscript along with their descriptions are listed in Table 4.

**Table 4:** List of abbreviations used in the manuscript

Abbreviation	Description
IPV	Intimate partner violence
IPVY	Preceding year intimate partner violence
IPVL	Lifetime intimate partner violence
PIPV	Physical intimate partner violence
SIPV	Sexual intimate partner violence
EIPV	Emotional intimate partner violence
CBIPV	Controlling behavior intimate partner violence
PIPVY	Preceding year physical intimate partner violence
SIPVY	Preceding year sexual intimate partner violence
EIPVY	Preceding year emotional intimate partner violence
PIPVL	Lifetime physical intimate partner violence
SIPVL	Lifetime sexual intimate partner violence
EIPVL	Lifetime emotional intimate partner violence
CBIPVL	Lifetime controlling behavior intimate partner violence
NDHS	Nepal Demographic Health Survey
BMI	Body mass index
AOR	Adjusted odds ratio
CI	Confidence Interval

## Data Availability

The datasets used in this study are publicly available upon request from Demographic Health Surveys Program.

## Appendix 1: Calculation of IPV Scores

**Table:**

IPV type	IPV form	Severity Score	Lifetime IPV		Preceding year IPV		
			Ever experienced?		Frequency		
			No (0)	Yes (1)	No (0)	Sometimes (1)	Often (2)
			0	1	0	1	2
Physical	Push you, shake you, or throw something at you?	1	0	1	0	1	2
	Slap you?	2	0	2	0	2	4
	Threaten or attack you with a knife, gun, or other weapon?	1	0	1	0	1	2
	Twist your arm or pull your hair?	1	0	1	0	1	2
	Punch you with his fist or with something that could hurt you?	3	0	3	0	3	6
	Kick you, drag you, or beat	3	0	3	0	3	6
	Choke you or burn you?	3	0	3	0	3	6
	Highest PIPVL score possible			14	Highest PIPVY score possible		28
Sexual	Physically force you to have sexual intercourse with him when you did not want to?	3	0	3	0	3	6
	Physically force you to perform any other unwanted sexual acts?	3	0	3	0	3	6
	Force you with threats or in any other way to perform sexual acts you did not want to?	3	0	3	0	3	6
	Highest SIPVL score possible			9	Highest SIPVY score possible		18
Emotional	Say or do something to humiliate you in front of others?	1	0	1	0	1	2
	Threaten to hurt or harm you or someone you care about?	2	0	2	0	2	4
	Insult you or make you feel bad about yourself?	1	0	1	0	1	2
	Highest EIPVL score possible			4	Highest EIPVY score possible		8
Controlling behavior	Jealous or angry if you talked to other men?	1	0	1	NA	NA	NA
	Frequently accused you of being unfaithful?	1	0	1	NA	NA	NA
	Does not permit you to meet your female friends?	1	0	1	NA	NA	NA
	Tried to limit your contact with your family?	1	0	1	NA	NA	NA
	Insisted on knowing where you were at all?	1	0	1	NA	NA	NA
	Highest CBIPVL score possible			5			

## Appendix 2: Designation of IPV intensity

**Table:**

		Intimate partner violence intensity			
IPV type	Scores range	None	Low	Moderate	High
Lifetime physical IPV (PIVPL)	0–14	0	1–4	5–9	10–14
Lifetime sexual IPV (SIPVL)	0–9	0	1–3	4–6	7–9
Lifetime emotional IPV (EIPVL)	0–4	0	1	2	3–4
Lifetime controlling behavior IPV (CBIPVL)	0–5	0	1	2–3	4–5
Preceding year physical IPV (PIPVY)	0–28	0	1–9	10–18	19–28
Preceding year sexual IPV (SIPVY)	0–18	0	1–3	4–11	12–18
Preceding year emotional IPV (EIPVY)	0–8	0	1–2	3–5	6–8
